# History of emergency medicine in Bhutan

**DOI:** 10.1186/s12245-024-00590-9

**Published:** 2024-01-22

**Authors:** Ugyen Tshering, Erol Kohli, Tashi Tenzin, Oriana Chen

**Affiliations:** 1 Emergency Department, Ballarat Base Hospital, Ballarat, VIC Australia; 2 Emergency Department, BronxCare Health System, Bronx, NY USA; 3 Department of Surgery, Jigme Dorji Wangchuck National Referral Hospital, Thimphu, Bhutan; 4 Emergency Department, Northeast Ohio Medical University, Rootstown, OH USA

**Keywords:** Emergency medicine, Bhutan

## Abstract

Emergency medicine in Bhutan has made significant progress in the past few decades and continues to evolve. In this article, we provide valuable insights into the history of emergency medicine at Jigme Dorji Wangchuck National Referral Hospital (JDWNRH) and in Bhutan and highlight some of the future challenges we face as we move forward to meet the demands of increased patient volume and complexity.

## Introduction

The Kingdom of Bhutan is a small (38,394 km^2^), land-locked country nestled in the eastern Himalayas, bordered by the larger nations of India to the south and China to the north with a population of 763,249 [1]. Bhutan’s strong international reputation lies with its focus upon “Gross National Happiness,” a concept pioneered by His Majesty Jigme Singye Wangchuck in 1974 and enshrined in the Bhutanese constitution in 2008 [2].

Gross National Happiness is a development philosophy which seeks to protect and nurture a nation’s ability to create and sustain happiness. Health is an important part of happiness, and for this reason, the Kingdom of Bhutan seeks to provide its citizens (and even its guests) with free healthcare. Bhutan’s ability to provide modern, scientifically based medicine is growing rapidly. Such growth is supported by a government that has invested heavily in a network of health units, district hospitals, regional referral centers, and dedicated staff [3]. Central to this network is the Jigme Dorji Wangchuck National Referral Hospital (JDWNRH) in Thimphu, which is Bhutan’s largest and most important hospital and serves as the tertiary care center for the entire country. Established in 1972 as a general hospital, it was rebuilt and expanded in 1994 and re-named as Jigme Dorji Wangchuck National Referral Hospital in honor of the third Druk Gyalpo, His late Majesty Jigme Dorji Wangchuck. This article aims to highlight the evolution of the specialty of emergency medicine in Bhutan and specifically in the Emergency Department (ED) at JDWNRH.

## Bhutan’s first emergency room (ER) at JDWNRH

In the late 1970s and early 1980s, emergency care for the acutely ill and injured patients in JDWNRH was provided in a single room staffed by the most junior medical officers who were responsible not only for the emergency room but also for patients on the inpatient medical wards and for walk-in patients visiting the hospital. While the ER was open 24 h a day, it lacked specialist services and advanced diagnostic capabilities.

## Casualty ward era

In the 1990s, the emergency room of JDWNRH was renamed as the casualty ward. It included a 4-bed casualty ward which served both medical and surgical cases. It was staffed by physicians who had no post graduate qualifications, or assistant clinical officers (non-physician providers) and nurses, but with access to on-call specialists. The casualty ward was poorly equipped, understaffed, unsupervised, and lacked staff that had specialized expertise in resuscitation and critical care.

There was no proper shift scheduling, and medical officers often worked 36-h shifts, while simultaneously attending to the admitted patients on all medical wards in the hospital, including the intensive care unit (ICU). In addition, the casualty ward also served as a clinic for all specialties with patients walking in for consultation and treatment.

The situation began to gradually improve in the early 2000s with the introduction of shift scheduling for all categories of staff that included nurses and physicians. However, on-duty medical officers continued to take care of patients on the floor, particularly after the clinics had closed. Initially, three shifts were established including 9:00 am to 3:00 pm, 3:00 pm to 9:00 pm, and 9:00 pm to 9:00 am.

## Formation of the emergency department

The emergency department was established in 2009 with Dr. Tashi Tenzin, a neurosurgeon, trained in India and Thailand, as the first head of the department. He provided much needed leadership and supervision and began to transform the department into a modern, functional ED. As more and more patients utilized the ED for emergency care, staffing for both doctors and nurses gradually increased. The ED was then staffed by medical officers with varying levels of ED experience and without specialty emergency medicine (EM) training. Although the quality of care started to improve, staffing remained a significant challenge and the scope of practice provided by the medical officers remained limited. By the end of 2009, the ED was relocated to the new hospital building and the functioning of ED changed considerably.

## Historic milestones of emergency medicine at JDWNRH and in Bhutan

1. In 2010, Health Volunteers Overseas (HVO), a United States (US)-based, non-profit organization, started sending volunteer emergency physicians from the US to JDWNRH to advance the practice of emergency medicine. This program has helped advance the practice of emergency care in the ED through lectures, hands-on training, simulation sessions, and informal bedside teaching. Their contributions have helped establish a culture of emergency medicine as a specialty in Bhutan. Furthermore, their volunteer work has substantially increased the scope of practice of emergency medicine at JDWNRH and has attracted a growing number of EM trained specialists to JDWNRH [4, 5].
2. In 2011, a 3-level triage system designed for resource-poor settings by the World Health Organization-International Committee of the Red Cross (WHO-ICRC) integrated triaging tool was introduced to improve patient safety through acuity assessment to assign patient treatment priorities [6]. Multiple studies have shown that such triage saves lives and reduces overall disability.
  The levels included the following: Immediate (Red), to be seen immediately; Urgent (Yellow), to be seen in < 60 min; and Standard (Green), to be seen within 120 min. Although patients are categorized into these 3 levels of triage, almost all patients were evaluated in less than an hour. This included the development of a triage area which is staffed by a nurse or emergency medical technician (EMT) trained and dedicated to performing this important role. Prior to this update, patients were seen not by acuity but by the order of their arrival to the ED.
3. In 2013, a collaboration between the Bhutan Foundation, a non-profit organization in the US, and the Ministry of Health began building Bhutan’s capacity in emergency medical services through training, supplying resources, equipment, and establishing the National Emergency Education Center (NEEC) located near the hospital at the Faculty of Nursing and Public Health (FoNPH) under the Khesar Gyalpo University of Medical Sciences (KGUMSB) which serves as the national emergency training center [5].
4. The first emergency medicine-trained physician joined the staff at JDWNRH in 2013, and between 2014 and 2022, the ED at JDWNRH had 2 full-time EM specialists. The third EM-trained physician joined Bhutan’s health service and has been leading the ED at Central Regional Referral Hospital in southern Bhutan. Since no EM-specific training program existed in Bhutan, physicians desiring to specialize in emergency medicine were required to go abroad to receive their training until June 2018. Until the mid-2022, Bhutan had only 3 trained emergency physicians.
5. A 4-year emergency medicine residency program was launched in July 2018 under the Faculty of Postgraduate Medicine (FoPGM), KGUMSB, with the inaugural class hosting two emergency medicine residents. The first 2 residents successfully graduated in July 2022 and are now leading the ED in Mongar Regional Referral Hospital in eastern Bhutan and Phuentsholing General Hospital in southern Bhutan.

## Pre-hospital care

Recognizing the importance of prehospital care, the Ministry of Health of the Royal Government of Bhutan established a centralized coordinating agency, called the Health Help Centre (HHC) to operate the prehospital Emergency Medical Services (EMS) system across the country in May 2011 [7]. Before the establishment of the HHC, EMS was predominantly a transportation service and not a prehospital medical service.

The ministry of health also established a national coordination and dispatch center located at HHC close to JDWNRH which is responsible for coordinating the dispatch of one hundred and ten land ambulances across the country. Each ambulance unit was staffed with an EMT and a driver with limited clinical skills. The system is activated by calling a toll-free national number (112) using computer aided dispatch. There is currently no priority dispatch system nor medical direction and oversight, which is under development.

The emergency medical technicians were recruited in 2011 as the HHC was established; however, they initially received only 8 weeks of training and thus had limited clinical skills and knowledge. In 2017, the Faculty of Nursing and Public Health (FoNPH) under the Khesar Gyalpo University of Medical Sciences of Bhutan (KGUMSB) initiated a formal 3-year diploma course for paramedics where the students spent their first year at the FoNPH and the remaining 2 years in the ED of JDWNRH and other departments of the JDWNRH. Since then, 3 batches of locally trained paramedics have graduated and are now the core pre-hospital service providers across Bhutan.

In November 2015, the Royal Bhutan Helicopter Service Limited was established and provides aeromedical evacuation of patients from across the country to JDWNRH and two other regional referral hospitals [8]. The helicopter medical crew, which comprises either a physician or a nurse or both, is under the medical control of the ED at JDWNRH and is financially supported by the ministry of health with 173 helicopter evacuations of patients made in the first year of its service.

Continued improvements in the aeromedical crew training and service led to the naming of helicopter aeromedical evacuation to Bhutan Emergency Aeromedical Retrieval (BEAR) in June 2017.

## Current practice at JDWNRH

The ED at JDWNRH now offers comprehensive emergency care and treats the highest level of critical injuries and illness, by acuity, while offering a broad range of acute care and specialty consultation. Patients have access to most major medical and surgical specialties by a dedicated team of physicians, nurses, and other health care professionals.

The ED has three acute care treatment areas comprising five resuscitation bays equipped with modern cardiorespiratory monitoring and support equipment and 13 acute care bays. Additionally, there is a consultation room where the walk-in patients are seen and treated. An EM attending physician is physically present in the ED from 9 am to 9 pm during the weekdays and provides consultative staffing in the evenings and weekends. The current staffing pattern provides a minimum of two medical doctors who are either emergency medicine residents or medical officers assisted by 4–6 nurses and 1–2 paramedics during each shift. ED physician staff include 2 emergency physicians, 8 emergency medicine residents, and 5 medical officers. Interns rotate through the ED with 6-week-long postings in the ED. Registration Data for 2022 showed a total of 42,665 ED visits.

## Current issues and future direction

While the emergency department has progressed from a simple receiving room in the initial stages to a department capable of providing complex medical care today, the burden of emergency care continues to rise along with patient and public expectations. Over the years, patient volume, medical and social complexity, and acuity of illness have substantially increased.

Lack of beds within the hospital has led to substantial ED overcrowding and long wait times for admissions. It has been shown that overcrowding is associated with adverse outcomes for patients and providers which include but are not limited to medical errors, increased morbidity and mortality, decreased patient satisfaction, job stress, and low staff morale.

The increasing patient volume and medical complexities we believe is related partly due to the fact that the JDWNRH hospital serves as the only tertiary care center for the entire country. The ED frequently receives critically ill patients who have traveled great distances, with very little prehospital care. Thus, we have had patients coming from rural and extremely remote regions of the country decompensate and ultimately die en route to the ED at JDWNRH.

Due to the limited outpatient clinic hours and ease of access to the ED which is open 24 h a day, the ED has become the public safety net leading to significant crowding which may interfere with providing care to the acutely ill who may need emergent lifesaving interventions. Initiatives to reduce ED utilization for non-emergent illness will need to focus on expanding outpatient clinic hours.

Furthermore, optimal staffing remains a significant challenge, and as the patient volume and services have increased, the number of EM specialists, medical officers, and nurses has not increased proportionately to meet the rising demands.

The establishment of our EM residency program in July 2018 has provided a source of locally trained EM specialists who can transform regional care in the same manner that the JDWNRH ED has done nationally.

## Conclusion

Emergency medicine in Bhutan has made significant progress in the past few decades and continues to evolve. We hope that this article will provide valuable insights into the history of EM at JDWNRH and in Bhutan and highlight some of the future challenges we face as we move forward to meet the demands of increased patient volume and complexity.

## Data Availability

Data sharing is not applicable to this article as no datasets were generated or analyzed during the current study.

## Abbreviations

JDWNRH: Jigme Dorji Wangchuck National Referral Hospital
ED: Emergency department
ER: Emergency room
ICU: Intensive care unit
EM: Emergency medicine
HVO: Health Volunteers Overseas
US: United States
WHO-ICRC: World Health Organization-International Committee of the Red Cross
EMT: Emergency medical technician
NEEC: National Emergency Training Center
FoNPH: Faculty of Nursing and Public Health
KGUMSB: Khesar Gyalpo University of Medical Sciences of Bhutan
FoPGM: Faculty of Postgraduate Medicine
HHC: Health Help Centre
EMS: Emergency medical services
BEAR: Bhutan Emergency Aeromedical Retrieval
